# Brain structure and structural basis of neurodegenerative diseases

**DOI:** 10.52601/bpr.2022.220013

**Published:** 2022-06-30

**Authors:** Jiawen Yang, Sen-Fang Sui, Zheng Liu

**Affiliations:** 1 Cryo-electron Microscopy Center, Southern University of Science and Technology, Shenzhen 518055, Guangdong, China; 2 Department of Biology, Southern University of Science and Technology, Shenzhen 518055, Guangdong, China; 3 State Key Laboratory of Membrane Biology, Beijing Advanced Innovation Center for Structural Biology, Beijing Frontier Research Center for Biological Structure, School of Life Sciences, Tsinghua University, Beijing 100084, China

**Keywords:** Brain atlas, Brain ultrastructure, Neurodegenerative disease, Parkinson’s disease, Alzheimer’s disease, Huntington’s disease

## Abstract

The brain is one of the most complex organs in nature. In this organ, multiple neurons, neuron clusters, or multiple brain regions are interconnected to form a complex structural network where various brain functions are completed through interaction. In recent years, multiple tools and techniques have been developed to analyze the composition of different cell types in the brain and to construct the brain atlas on macroscopic, mesoscopic, and microscopic levels. Meanwhile, researchers have found that many neuropsychiatric diseases, such as Parkinson’s disease, Alzheimer’s disease, and Huntington’s disease, are closely related to abnormal changes of brain structure, which means the investigation in brain structure not only provides a new idea for understanding the pathological mechanism of the diseases, but also provides imaging markers for early diagnosis and potential treatment. This article pays attention to the research of human brain structure, reviews the research progress of human brain structure and the structural mechanism of neurodegenerative diseases, and discusses the problems and prospects in the field.

## INTRODUCTION

The brain is one of the most important organs that are responsible for the regulation of body function. The human brain consists of three parts: the forebrain, midbrain, and hindbrain (Volpe 2009). As the largest part of the human brain, the function of the cerebrum is higher such as thinking and movement. It is divided into the frontal lobe, temporal lobe, parietal lobe and occipital lobe (Saygin and Sereno 2008). The surface of the cerebrum is called the cortex which contains gray matter (the main components of the central nervous system, CNS), while the gray matter is interconnected to other brain areas by axons (white matter) (Henmar* et al.* 2020). The location of the midbrain is between the cerebral cortex and the hindbrain, it participates in controlling body movements while acting as a relay station for the visual and auditory systems (Knudsen 2020; Wohlgemuth and Moss 2016). The adjustment and coordination of movement, posture, and balance are inseparable from the cerebellum, while the brain stem is responsible for maintaining individual life. Together, they form the hindbrain (Roostaei* et al.* 2014). As the center of the nervous system, the brain is important and unique to individuals, it can provide coherent control over the actions of individuals, it is essential for perception, motor control, activation, emotion, speaking, learning and memory, as well as balance regulation (Carew 2000).

There are many kinds of diseases related to the brain, such as brain tumors, brain trauma, cerebrovascular diseases, inflammatory diseases, inherited metabolic diseases, and neurodegenerative diseases. Neurodegeneration is the process of neuronal structure and function loss, which can be found in the brain at different levels of neuronal circuitry (Chen* et al.* 2016). It can lead to many neurodegenerative diseases such as Parkinson's disease (PD), Alzheimer's disease (AD) and Huntington's disease (HD). These diseases are various in their pathophysiology, some of them cause memory and cognitive impairments, and others influence the ability to move, speak and breathe (Abeliovich and Gitler 2016; Canter* et al.* 2016; Taylor* et al.* 2016; Wyss-Coray 2016). With the deepen research on neurodegenerative diseases, more and more factors such as oxidative stress, mitochondrial dysfunction, excitotoxins, immune inflammation, calcium imbalance, and cell apoptosis (Xue 2015) are found to have a connection with the occurrence and development of neurodegenerative diseases. It is estimated that 50 million people all over the world suffer from neurodegenerative diseases, and this figure will increase to 115 million by the year 2050 (Livingston* et al.* 2020; Rodríguez* et al.* 2015). As a result, it is crucial to understand the mechanisms of each disease, find the hallmarks for early diagnosis, and discover the potential drug targets, all are contributing to effective treatments.

Many researchers focus on the structure of the brain to understand the mechanism of the brain disease and provide effective drugs or methods of therapy. On the one hand, with the development of the technique of sample preparation and imaging data collection and analysis, the ultrastructure of the key regions, organelles, or chemical substances in the healthy and disease brain has been revealed gradually. On the other hand, the development of cryo-electron microscopy (cryo-EM) and direct electron detectors enables researchers to solve the structures of pathogenic proteins. In addition, the concept of the human connectome (the connection matrix of the brain) has been formally proposed in 2005 (Sporns* et al.* 2005). Since 2009, the National Institutes of Health (NIH) has begun to fund the Human Connectome Project whose aim is to draw different atlas of human brain functions and structures in living organisms with different brain imaging techniques (Van Essen and Ugurbil 2012). This project plans to describe the human brain structure network atlas comprehensively and meticulously on macroscopic, mesoscopic, and microscopic levels, and further explore the neuronal connection regulation of the brain. The construction of the human brain connectome is difficult due to a large number of different types of neuronal cells, trillions of synapses, the complex connection patterns, and dynamic changes between their construction in the human brain, however, it is also significant because it helps us to understand the organization of the brain, changes the way we study the brain for a long time, considers the brain as a complex unity of neurons that are interconnected instead of a huge number of discrete anatomical units or a collection of chemical substances (Lehrer 2009).

This review focuses on the research progress of brain structure and the key structures of the brain with PD, AD, and HD to understand the structural basis of neurodegenerative diseases. As there are many different techniques are studying the brain structure on macroscopic, mesoscopic, and microscopic levels, it is necessary to know about the advantages and disadvantages of each technique. Combined with the atomic resolution structure of the pathogenic proteins *in vitro* and the ultrastructure of the key regions and organelles *in situ*, the brain atlas has great potential to make contributions to mechanism understanding, early diagnosis, and drug discovery of neurodegenerative as well as other types of brain disease.

## BRAIN ATLAS ON DIFFERENT LEVELS

The human brain structure can be investigated from the microscale, mesoscale, and macroscale which represent brain regions and pathways, cortical mini-columns and their connection patterns as well as single neurons and synapses respectively (Sporns* et al.* 2005). Researchers in different fields use different techniques to study the structure of the brain at different levels, and with the improvement of these techniques, there has been the brain atlas on different levels.

### Macroscopic and mesoscopic atlas of brain

As one of the most popular tools to study the brain structure on the macroscopic level, magnetic resonance imaging (MRI) can provide comprehensive, multi-parameter information about the anatomy, function, and metabolism of the brain (Yousaf* et al.* 2018). It can measure grey matter volume, cortical thickness, and hippocampus volume (Chandra* et al.* 2019) and is usually used in clinical diagnosis. In 2016, researchers from Washington University School of Medicine in St. Louis used MRI to establish a detailed atlas of the human cerebral cortex which is involved in sensory perception, attention, and distinct human functions such as speaking, tool use, and abstract thinking (Glasser* et al.* 2016). This atlas divides the left and right hemispheres into 180 regions based on physical differences (such as the thickness of the cortex), functional differences (such as which regions respond to language stimuli) and brain region connection differences. Among these regions, some of them are obviously involved in specific activities, such as vision and movement control, and most regions are identified as multi-functional because they coordinate information from many different signals instead of just doing one thing. It is clear that the best spatial resolution of clinical images of the neuronal system can be provided by MRI (Femminella* et al.* 2018), however, the spatial specificity and temporal response of the alternative signal which reflects the mass activity of neurons measured by MRI are limited by both physical and biological elements (Logothetis 2008). In addition, the resolution of MRI is too low to study the brain structure in detail.

Light microscope (LM) serves as the basis to study the brain structure on mesoscopic level. In the year 2010, a micro-optical sectioning tomography (MOST) system based on the light microscope, microtome, and image recorder was advanced by Luo’s team. With this system, micrometer-scale tomography of a whole mouse brain was provided and a three-dimensional (3D) structural data set of a Golgi-stained whole mouse brain at the neurite level (Li* et al.* 2010) were obtained. This 3D atlas clearly shows the morphology, localization, and interconnectivity of neural circuits in all places of the brain, which is in favor of studying the function of nervous systems as well as the mechanisms of nervous system diseases. A few years later, they developed the brain-wide positioning system (BPS) to automatically dissect and locate neurons marked by cell structure with single cell resolution (Gong* et al.* 2016) and obtained the high-resolution continuous neural projection (Gong* et al.* 2016) and projection patterns of the cholinergic neurons (Li* et al.* 2018b). Recently, they further developed high-definition fluorescent micro-optical sectioning tomography (HD-fMOST) and raised the quality of the whole brain optical imaging to high definition (Zhong* et al.* 2021). This is currently the fastest technology for realizing whole-brain optical imaging with similar voxel resolution, which is expected to play an important role in standardized and large-scale brain science research.

### Key ultrastructure and microscopic atlas of brain

**Figure 1:**
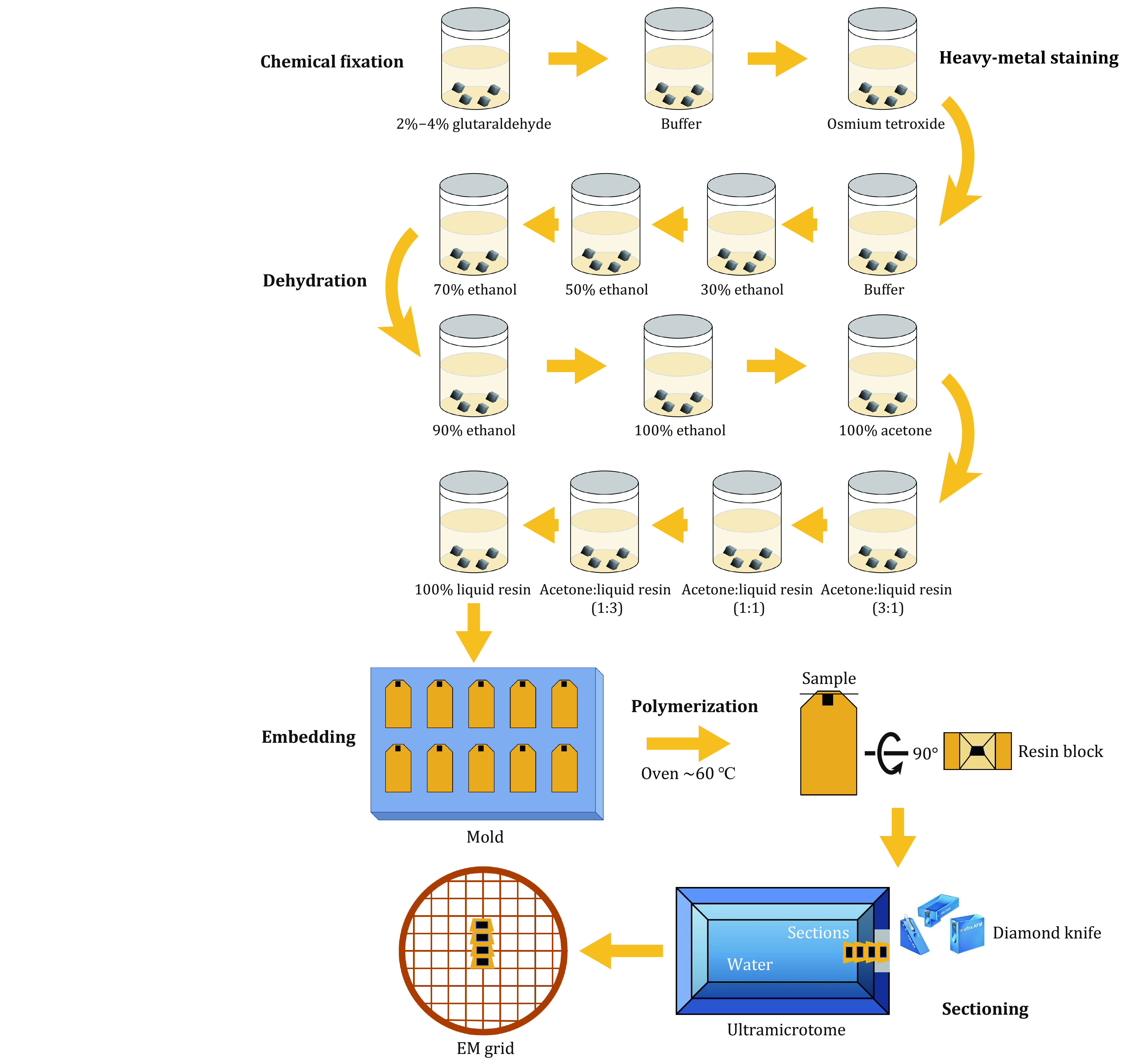
Basic sample preparation workflow. It shows the main processes of sample preparation, starting from chemical fixation and ending with ultra-sectioning

Electron microscopy (EM) is widely used in brain structure study on the microscopic level due to its atomic resolution. The electron microscope was invented by Ernst Ruska in the 1930s (Shampo and Kyle 1997) and the basic problems of imaging biological materials by EM are solved depending on the development of several key techniques. The improvement of the ultramicrotome (Porter and Blum 1953; Sjostrand 1953) with glass (Latta and Hartmann 1950) and diamond knives (Fernandez-Moran 1953) enables the preparation of sufficiently thin specimens (Al-Amoudi* et al.* 2004). The basic sample preparation workflow includes chemical fixation, heavy-metal staining, dehydration, embedding, polymerization, and sectioning (Fig. 1). This is the main method for the identification of the ultrastructure of the brain.

As an efficient tool for the characterization of brain ultrastructure, EM solves many pathological problems related to diseases. For example, various types of cell death including necrosis, ferroptosis and autophagy, and different degrees of axon degeneration were observed under the condition of striatum injury in the acute phase of cerebral hemorrhage (ICH) (Li* et al.* 2018a). These findings provide valuable information for ICH pathology. Another example is the observation of the ultrastructure related to the blood-brain barrier (BBB). BBB refers to the barrier between blood plasma and brain cells formed by brain capillary walls and glial cells and the barrier between plasma and cerebrospinal fluid formed by the choroid plexus, which can prevent certain substances (mostly harmful) enter the brain tissue from the blood (Daneman and Prat 2015). Michalski *et al.* thought that the application of the embolic middle cerebral artery occlusion (eMCAO) model led to changes in the structure of blood vessels which were reflected in the extravasation of FITC-albumin (Michalski* et al.* 2010), however, there was no observation of BBB breakdown on another hemisphere because there was no FITC-albumin leakage in the particular areas in later research (Krueger* et al.* 2013). Ando *et al.* indicated that the denser structure of glycocalyx in the brain was linked to endothelial protection and might be a key element of the BBB because there was still endothelial glycocalyx within cerebral capillaries under the condition of lipopolysaccharide-induced vascular injury (Ando* et al.* 2018). The above examples illustrated that EM has made a great contribution to the study of the mechanism of brain diseases.

**Figure 2:**
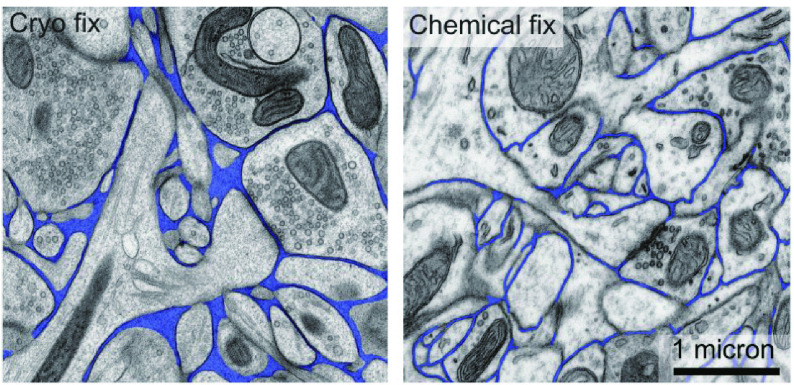
Differences of ultrastructure between the cryo-fixed and chemical-fixed synaptic samples. The observation of cryo-fixed and chemical-fixed neuropil from the cerebral cortex of an adult mouse indicates that there is a reduction of extracellular space (blue) after chemical fixation. Adapted from Korogod *et al.* (2015) with permission

To avoid the artifacts caused by the heavy metal staining, rapid freezing was first used as the initial step in an EM preparation approach in 1957 (Muller 1957). EM samples prepared by traditional chemical fixation methods are prone to artificial artifacts such as biofilm shrinkage, matrix loss, cell component extraction, and abnormal organelles (Bullen* et al.* 2014). Low-temperature sample preparation method can prevent samples from chemical damage in conventional sample preparation, making the ultrastructure of the cell closer to its native state. Physical fixation such as high pressure freezing (HPF) and plunge freezing is mainly used in the preparation of low-temperature samples. On the one hand, the HPF fixation is a physical fixation method that uses the special properties of water to instantly vitrify the sample to achieve freezing fixation (Zhang 2016). Compared to the cryo-fixed sections, there were enlarged ventricles and a clear reduction as well as significantly higher synapse density in chemical-fixed sections (Fig. 2) (Korogod* et al.* 2015). However, the ice crystal pollution should be avoided during the preparation of low-temperature samples.

On the other hand, Liu *et al.* used plunge freezing and cryo-electron tomography (cryo-ET) to identify type-A GABA receptors (GABA_A_Rs) in inhibitory synapses and determined their native structure at 19 Å resolution. It reveals a hierarchical organization of GABA_A_Rs whose function is associated with the presynaptic neurotransmitter release (Liu* et al.* 2020). This is the first attempt to use cryo-ET to analyze the location of receptor proteins, providing the basis for *in situ* high-resolution analysis of receptor molecules and other proteins, as well as the mechanism and development of the corresponding drugs. Avoiding the steps of sample sectioning seems to make it easier than other methods, however, there is an obvious limitation of the thickness of the sample.

With the development of EM technology, there have been different methods of sample preparation. A new sample preparation method called brain-wide reduced-osmium staining with pyrogallol-mediated amplification (BROPA) was used for the reconstruction of neural circuits spanning an entire mouse brain combined with serial block-face electron microscopy (SBEM) (Mikula and Denk 2015). This method overcame the difficulty that the tissue preparation of neural circuits on large scale was only suitable for small samples (Briggman* et al.* 2011; Jarrell* et al.* 2012), as well as avoided the damage on the boundary of the fractional plane between the dividing tissue. Recently, a group of neuroscientists in Harvard University and Google engineers completed the first diagram of the human brain connectome. The human surgical sample from the temporal lobe of the cerebral cortex was collected, after heavy metal staining and resin embedding, a 1-mm^3^ volume block was cut into more than 5000 slices whose thickness was 30 nm. The collection of sections was with the help of an automatic tape-collecting ultramicrotome (ATUM) and these sections were observed with a high-speed multi-beam scanning electron microscope (mSEM). After alignment, segmentation, agglomeration, synapse detection and classification, and error correction, the brain sample was analyzed on macroscopic and microscopic levels (Shapson-Coe *et al*. 2021). This research fills the gap in the 3D ultrastructure of the human brain and is a structural basis of the mechanism study of brain diseases. However, huge data storage and long-term data collection are still major issues that need to be improved.

## STRUCTURAL BASIS OF NEURODEGENERATIVE DISEASES

Neurodegenerative diseases such as Parkinson’s disease (PD), Alzheimer’s disease (AD), and Huntington’s disease (HD) are increasingly considered to have similar cellular and molecular mechanisms, including protein aggregation, mitochondria dysfunction, and inclusion body formation (Ross and Poirier 2004). It is difficult for the treatment of advanced neurodegenerative diseases due to the irreversibility of neuron death. As a result, the hallmarks for early diagnosis and understanding the structural mechanism of the diseases are two key factors of the therapy. The researches about the understanding of the structure basis of PD, AD and HD will be introduced respectively in more detail.

### Parkinson’s disease

Parkinson’s disease (PD) is the second-most common neurodegenerative disorder which can cause pathological static tremor, rigidity, bradykinesia, depression, anxiety, sleep disturbance and cognitive impairment (Kalia and Lang 2015). In terms of pathology, PD is characterized by a gradual decrease of dopaminergic neurons in the substantia nigra pars compacta and nonspecific cortical atrophy with sulcal widening and enlargement of the lateral ventricles (Balestrino and Schapira 2020). Although the aetiology of PD in most patients is unclear, different pathological causes have been identified such as the accumulation of misfolded α-synuclein (α-Syn), the mutation of Leucine-rich repeat kinase 2 (LRRK2) and other impairment of mitochondrial function (Balestrino and Schapira 2020; Monfrini and Di Fonzo 2017; Schapira* et al.* 1989).

**Figure 3:**
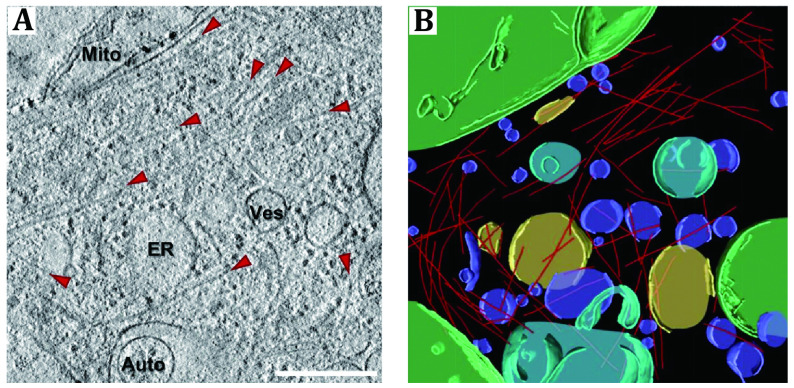
Cryo-ET imaging of neuronal α-Syn aggregates. **A** A tomographic slice shows the intracellular organization of a p62-RFP-expressing neuron, mitochondria (Mito), vesicles (Ves), endoplasmic reticulum (ER), autophagosome (Auto) and α-Syn fibers (labelled by red arrows) can be seen. Scale bar: 350 nm. Auto; autophagosome, ER; endoplasmic reticulum, Mito; mitochondrion, Ves; vesicles. **B** 3D rendering of the tomogram (panel A) shows the location and organization of α-Syn fibrils (red), an autophagosome (cyan), ER (yellow), mitochondria (green), and various vesicles (purple). Adapted from Trinkaus *et al.* (2021) with permission

*SNCA* is linked to PD and it is the gene encoding for α-Syn (Gómez-Benito* et al.* 2020). *A53T* was the first pathogenic *SNCA* mutation identified which causes α-Syn to misfold and easily aggregate than the wild-type (Polymeropoulos* et al.* 1997). The protein α-Syn has been found to be a major component of Lewy bodies and is considered to play a central role in their formation (Goedert* et al.* 2013). α-Syn aggregation has a close connection with PD and the pathological α-Syn is considered with amyloid fold (Henderson* et al.* 2019; Ke* et al.* 2020). Shahmoradian *et al.* using correlative EM on chemically fixed PD brain tissue revealed that Lewy pathology consists of vesicular structures interspersed with filaments and abundant tubulovesicular structures and suggested that cellular membranes with fibrillar α-Syn density were the main element of Lewy bodies (Shahmoradian* et al.* 2019). Trinkaus *et al.* studied the α-Syn inclusions formed in their cellular system by cryo-focused ion beam (cryo-FIB) milling and cryo-ET and demonstrated that neuronal α-Syn fibrils can form α-Syn inclusions with membranous organelles but doesn’t interact with membranes directly (Fig. 3) (Trinkaus* et al.* 2021).

Mutations in LRRK2 are the key element of familial Parkinson’s disease (Chan and Tan 2017). LRRK2 is a large 288 kDa protein and an N-terminal half containing repeating motifs (armadillo, ankyrin, and leucine-rich repeats), a C-terminal half composed of kinase and GTPase, and two other domains (C-terminal of ROC (COR), and WD40) are the key parts of LRRK2 (Deniston* et al.* 2020; Kalia and Lang 2015). The 14 Å resolution *in situ* cryo-ET structure of full-length human LRRK2 bound to microtubules was obtained which contains all regions related to PD mutations with the help of the cryo-correlative light and electron microscopy (cryo-CLEM) and sub-tomogram analysis (Watanabe* et al.* 2020). Meanwhile, Deniston *et al.* reported a 3.5 Å cryo-EM structure of the catalytic half of LRRK2 and built an atomic model using the cryo-ET structure above (Deniston* et al.* 2020). In summary, the study of LRRK2 structure lays the foundation for a promising future of mechanism in PD research.

In addition, mitochondrial dysfunction is a key mechanism of PD. Different genes related to PD regulate mitochondrial functions. PTEN-induced putative kinase 1 (PINK1) (Schubert* et al.* 2017) and Parkin (Trempe* et al.* 2013) interact with each other to control mitochondria quality. PINK1/Parkin pathway acts as a hub for mitochondrial maintenance, PINK1 can accumulate on the outer surface of the damaged mitochondria so that leads to the recruitment of Parkin, an E3 ubiquitin ligase, to activate mitochondrial autophagy (Balestrino and Schapira 2020). Gan *et al.* used crystallography and cryo-EM to indicate the activation system of PINK1. The non-phosphorylated PINK1 monomers accumulate on the outer surface of depolarized mitochondria and form PINK1 dimers, which can be trans-autophosphorylated with the conformational changes of α-C helix. This can lead to the dissociation of PINK1 dimers and enable the recruitment of ubiquitin and the ubiquitin-like domain of Parkin which can result in mitophagy (Gan* et al.* 2022).

### Alzheimer’s disease

Alzheimer’s disease (AD) is the most common neurodegenerative disease which can cause the loss of memory, disorders of time and space orientation, cognitive dysfunction, behavioral abnormalities, and social disturbances (Schellenberg and Montine 2012). Recent epidemiological data indicate that there are 9.83 million cases of AD patients in the population over 60 years old in China (Jia* et al.* 2020). MRI images indicate that there is a reduction in hippocampal volume and cerebral cortex atrophy in individuals which is supposed to be the responsibility of dementia (Femminella* et al.* 2018). In terms of pathology, the extracellular accumulation of amyloid (Aβ) plaques and intraneuronal deposits of neurofibrillary tangles (NFTs) are the two key factors of AD characterization (Rastogi* et al.* 2021).

The amyloid hypothesis of AD considers that the aggregation of Aβ peptides which is the product of the abnormal cleavage of the amyloid precursor protein (APP) forms insoluble Aβ plaques and further drives the incidence of AD (Selkoe and Hardy 2016). Observation of Aβ plaques in human cases of AD (El Hajj* et al.* 2019) is consistent with previous studies (Nixon* et al.* 2005; Sanchez-Varo* et al.* 2012). In 2017, Gremer *et al.* used cryo-EM to represent a 4 Å resolution Aβ (1–42) fibril structure and illustrated that the regular helical symmetry had a direct influence on the mechanism of fibril elongation and led to distinct binding sites for monomeric Aβ (Gremer* et al.* 2017).

**Figure 4:**
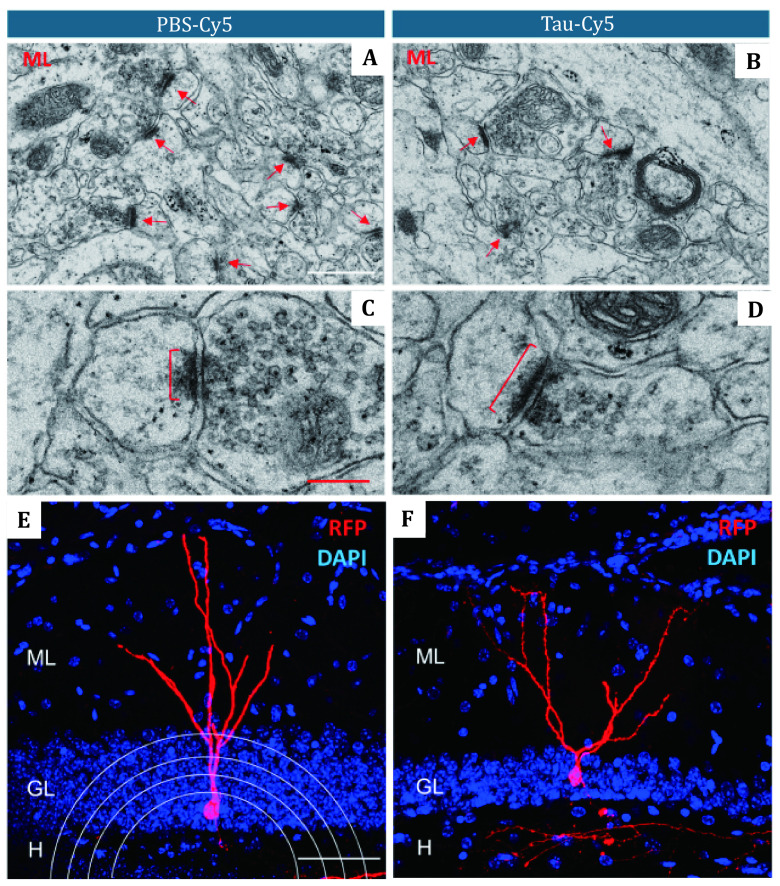
The soluble Tau can alter hippocampal granule neurons synapses and newborn granule neurons. Compared to the control group (**A**, **C** and **E**), the soluble Tau (**B** and **D**) has long-term influences on afferent synapses in the molecular layer (ML) with the lower density of synapses (red arrows), higher postsynaptic density (brackets). It also changes the morphology of newborn granule neurons (**F**). Red scale bar: 200 nm. White scale bar: 50 μm. ML: molecular layer; GL: granular layer; H: hilus. Adapted from Bolós *et al.* (2017) with permission

The misfolding and aggregation of Tau protein is also a key mechanism of AD. Tau is one of the microtubule-associated proteins (MAP) that play important roles in microtubule regulation to ensure cytoskeletal organization and trafficking (Aamodt and Williams 1984), its hyperphosphorylation can lead to NFTs, which leads to cognitive dysfunction (Kim* et al.* 2020). Bolós *et al.* indicated that Tau could impair the plasticity of hippocampal granule neurons and change the morphology of newborn granule neurons (Fig. 4) (Bolós* et al.* 2017). Tau not only can aggregate themselves but also cause further misfolding in a process called templated misfolding (Brunello* et al.* 2020) while there is more and more evidence suggesting that inclusions of misfolded pathological proteins spread along neuronal connection areas in neurodegenerative disease (Jucker and Walker 2013). Fitzpatrick *et al.* completed the atomic characterization of Tau filaments composing Tau inclusions from AD and considered that it was essential for the assembly of Tau in AD to form the ordered core of pairs of protofilaments comprising residues 306–378 (Fitzpatrick* et al.* 2017).

### Huntington’s disease

**Figure 5:**
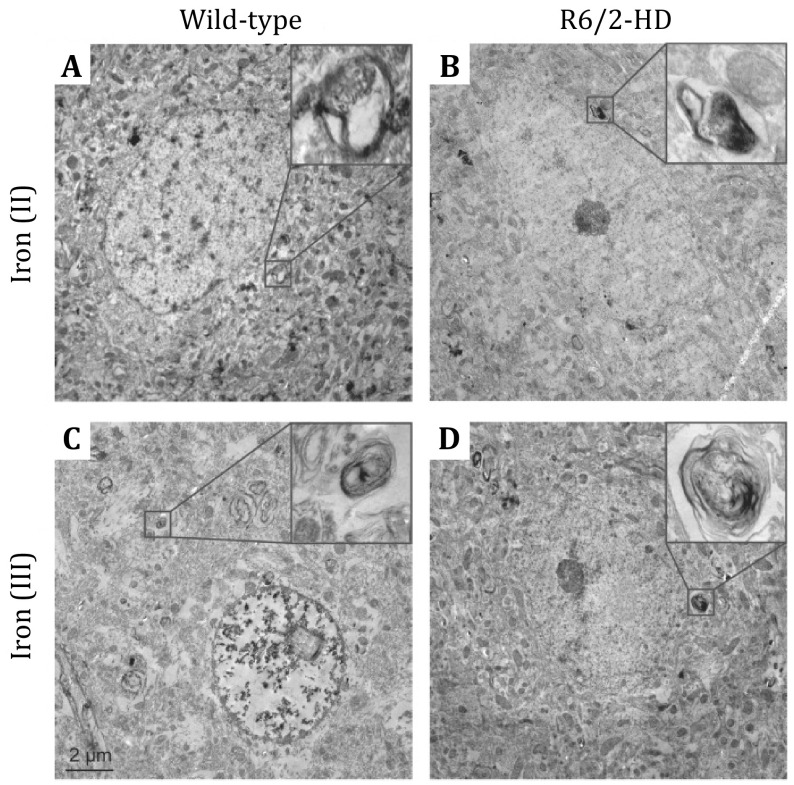
Iron accumulation in R6/2 HD striatal neurons. There was abundant iron (II) staining in the cytoplasm of striatal neurons with the complicated membrane (**A** and **B**), while there were no differences in iron (III) in the striatum between WT and HD mice (**C** and **D**). Adapted from Chen *et al.* (2013) with permission

Huntington’s disease (HD) is an autosomal-dominant neurodegenerative disorder that can cause motor symptoms, cognitive dysfunction, and psychiatric disorder. The typical symptom of HD is chorea, affected by the disease, the HD patients will be temporarily uncontrollable grimacing, nodding, and finger beating, and even worse, typical dance-like involuntary movements, and difficulty swallowing (Burgunder 2015). An MRI of HD shows damaged caudate and global atrophy dominated by the frontal lobe (Goh* et al.* 2018). The exact mechanism of HD pathogenesis is currently unknown, but the pathogenic gene *HTT* has been recognized (Nakamura 1993). A mutation in the *HTT* gene can cause an abnormal expansion of a polyglutamine (polyQ) repeat at the amino terminus of Huntingtin (HTT) protein, which results in HD (Finkbeiner 2011; Nakamura 1993). HTT is a 348 kDa protein that plays important role in embryonic development and various cellular activities (Saudou and Humbert 2016). On the one hand, the cryo-EM structure of full-length human HTT in a complex was demonstrated (Guo* et al.* 2018). On the other hand, there are also many researchers focusing on protein aggregation *in situ*. Li *et al.* used electron microscopic immunocytochemistry to identify the HTT aggregation in HD mouse model (Li* et al.* 2001). In 2017, Bäuerlein *et al.* found that polyQ inclusions are composed of amyloid-like fibrils interacting with cellular endo-membranes, especially the endoplasmic reticulum (ER) (Bäuerlein* et al.* 2017). Their results suggested the previous study that wild-type HTT has an interaction with cellular membranes (Kegel-Gleason 2013), and fibrils of polyQ-expanded HTT exon 1 and other amyloids can cause membrane disruption (Milanesi* et al.* 2012; Pieri* et al.* 2012). In addition, HTT expression can lead to the accumulation of brain iron in human HD (Rosas* et al.* 2012), and indicates it is ferrous (II) iron that contributes to this accumulation rather than ferric (III) iron (Fig. 5) (Chen* et al.* 2013).

## CONCLUSION

The brain is the most complex and delicate system in the world, therefore, the structural and functional research of the brain has always been the most challenging subject. Many researchers are utilizing the structure determination of the brain to understand the mechanism of its function, development and plasticity.

With the development of techniques such as MRI, LM, and EM, the structure of the key regions like organelles and proteins are studied on macroscopic, mesoscopic and microscopic levels. MRI is a type of technique that the signal generated by the nuclei through resonance is reconstructed to form an image in a specific field (Santos Armentia* et al.* 2019). Due to its clearer imaging, brain MRI can provide more information than other brain imaging technology and it is widely used in the examination of the whole brain. There are many advantages of brain MRI such as high sensitivity, multi-directional slices, multi-parameter imaging and high accuracy, so that many researchers use it to study the brain structure on the macroscopic level. Since the first MRI atlas of the human cerebral cortex was constructed in 2016 by Glasser and colleagues (Glasser* et al.* 2016), MRI technique has gradually been used in brain atlas construction (Kim* et al.* 2021), and in 2022, a spatiotemporal (four-dimensional) infant-dedicated brain MRI atlas was constructed based on the UNC/UMN Baby Connectome Project (BCP) dataset (Chen* et al.* 2022). The macroscopic MRI brain atlases have great potential for disease therapy, however, the finely detailed brain ultrastructure cannot be obtained by MRI, thus other types of techniques such as LM and EM are also important in brain structure study. Li *et al.* constructed the mesoscopic atlas of the mouse brain at the neurite level (Li* et al.* 2010), Oh *et al.* obtained a mesoscale connectome of the mouse brain by fluorescent LM (Oh* et al.* 2014), and Friedmann *et al.* mapped the mesoscale axonal projections of the mouse brain by light-sheet microscope (Friedmann* et al.* 2020). These results provide information about neuron arrangement and connection as well as can be the reference for the treatment of cranial nerve diseases.

The development of modern technology and biomedicine has brought about comprehensive technological progress in the field of clinical medicine, which promotes the innovation and improvement of the medical level. The conception of precision medicine is proposed to use genomic information from individual diseases to guide accurate diagnosis and medication (König* et al.* 2017). The practice of precision medicine relied on the understanding of disease mechanisms and the identification of pathogenic targets. The abnormal structure of the pathogenic proteins such as LRRK2, Tau, HTT and the aggregation of specific proteins such as polyQ and α-Syn can be the hallmarks of neurodegenerative diseases and have great potential in precision medicine. Although there has already been some macroscopic, mesoscopic atlas of the brain, it is important to improve the microscopic atlas of the brain as well as the brain with neurodegenerative disease, which will contribute to the improvement of target drug design and disease treatment.

## Conflict of interest

Jiawen Yang, Sen-Fang Sui and Zheng Liu declare that they have no conflict of interest.
